# Microbial and enzymatic changes in cigar tobacco leaves during air-curing and fermentation

**DOI:** 10.1007/s00253-023-12663-5

**Published:** 2023-07-17

**Authors:** Qing Zhang, Guanghui Kong, Gaokun Zhao, Jun Liu, Honggang Jin, Zhihua Li, Guanghai Zhang, Tao Liu

**Affiliations:** 1 College of Agriculture and Biotechnology, Yunnan Agricultural University, Kunming, 650201 Yunnan China; 2 Yunnan Academy of Tobacco Agricultural Sciences, Kunming, 650021 Yunnan China; 3 Raw Materials Department of HongYun HongHe Tobacco (Group) Limited Liability Company, Kunming, 650221 Yunnan China

**Keywords:** Cigar tobacco leaf, Microbial community, Enzyme activity, Air-curing, Fermentation

## Abstract

**Abstract:**

Metabolic enzyme activity and microbial composition of the air-curing and fermentation processes determine the quality of cigar tobacco leaves (CTLs). In this study, we reveal the evolution of the dominant microorganisms and microbial community structure at different stages of the air-curing and fermentation processes of CTLs. The results showed that the changes in metabolic enzymes occurred mainly during the air-curing phase, with polyphenol oxidase (PPO) being the most active at the browning phase. *Pseudomonas*, *Bacteroides*, *Vibrio*, *Monographella*, *Bipolaris*, and *Aspergillus* were the key microorganisms in the air-curing and fermentation processes. Principal coordinate analysis revealed significant separation of microbial communities between the air-curing and fermentation phases. Redundancy analysis showed that bacteria such as *Proteobacteria*, *Firmicutes*, *Bacteroidota*, and *Acidobacteriota* and fungi such as *Ascomycota* and *Basidiomycota* were correlated with enzyme activity and temperature and humidity. Bacteria mainly act in sugar metabolism, lipid metabolism, and amino acid metabolism, while fungi mainly degrade lignin, cellulose, and pectin through saprophytic action. Spearman correlation network analysis showed that *Firmicutes*, *Proteobacteria*, and *Actinobacteria* were the key bacterial taxa, while *Dothideomycetes*, *Sordariomycetes*, and *Eurotiomycetes* were the key fungal taxa. This research provides the basis for improving the quality of cigars by improving the air-curing and fermentation processes.

**Key points:**

• *Changes in POD and PPO activity control the color change of CTLs at the air-curing stage.*

• *Monographella, Aspergillus, Pseudomonas, and Vibrio play an important role in air-curing and fermentation.*

• *Environmental temperature and humidity mainly affect the fermentation process, whereas bacteria such as Proteobacteria, Firmicutes, Bacteroidota, and Acidobacteriota and fungi such as Ascomycota and Basidiomycota are associated with enzyme activity and temperature and humidity.*

**Graphical Abstract:**

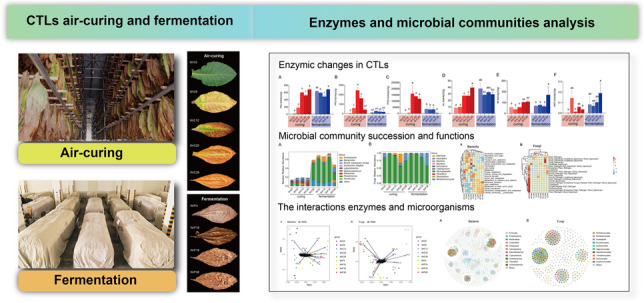

**Supplementary Information:**

The online version contains supplementary material available at 10.1007/s00253-023-12663-5.

## Introduction

Cigars are made from freshly picked cigar tobacco leaves (CTLs) that are processed by air-curing and fermentation. The modulated CTL is highly sought after and loved for its unique flavor and aroma characteristics (Viola et al. 2016). Modulation, which is divided into two stages, air-curing and fermentation, is performed mainly to improve the quality of tobacco through complex dynamic changes in metabolic enzyme activity and microbial community composition. Enzymes can improve the appearance of tobacco by breaking down starch, sugars, proteins, pigments, and other substances and increasing the levels of aroma-causing components (Zheng et al. 2022). Microorganisms produce small-molecule metabolites by secreting metabolism-related enzymes that breakdown and use organic substrates such as sugars, proteins, and fats to promote growth and reproduction (Liu et al. 2021). The metabolic activities of the microbial community composition, including carbohydrate degradation, chlorogenic acid degradation, protein degradation, fatty acid and lipid biosynthesis, amino acid biosynthesis, and aromatic compound biosynthesis, are the main reasons for the distinctive flavor of CTLs (Banožić et al. 2020; Liu et al. 2021; Wang et al. 2022; Zheng et al. 2022). The carbonyl groups in the molecular structure of aldehydes and ketones give tobacco a distinctive aroma; esters provide floral, fruity, and sweet aromas; pyrazine provides nutty and roasted aromas; and furan compounds provide a distinctive caramel aroma (Chung et al. 2020; Piornos et al. 2020).

The dominant microorganisms of CTLs play a major role in the degradation of aromatic compounds, hydrocarbons, aliphatic compounds, and lignin, and the structure of the microbial community composition varies from region to region, making cigar tobacco flavor highly variable. The dominant microorganisms involved in the fermentation of tobacco are mainly bacteria, with molds and yeasts also participating in the fermentation process (Frankenburg 1947). *Bacillus*, *Pseudomonas*, *Enterobacter*, *Sphingomonas*, *Pantomonas*, and *Methylobacterium* are the main bacterial genera involved in the fermentation of CTLs (Huang et al. 2010; Zhou et al. 2020). During fermentation, *Pseudomonas* can effectively degrade nicotine (Zhong et al. 2010; Liu et al. 2015a, b; Wang et al. 2023), and *Bacillus* can breakdown large molecules such as lignin and carotene by secreting related enzymes to produce small aromatic substances (Dai et al. 2020), resulting in the improved appearance and aroma of CTLs, among other effects. During bacterial fermentation, *Proteobacteria* and *Actinomycetes* degrade large molecules such as cellulose, pectin, and starch to small molecules such as glucose, fructose, and maltose (Costa et al. 2020), while fungal fermentation is mainly putrefactive (Liu et al. 2021). Yeasts, *Staphylococcaceae*, and *Lactobacillus* act mainly in the early stages of tobacco fermentation (Di Giacomo et al. 2007). Moreover, there is a correlation between the microbial community composition and the ambient temperature and humidity during the modulation process (Zhao et al. 2022).

To date, studies on the microbial communities of CTLs have focused mainly on the screening of functional microorganisms in the fermentation stage and the analysis of the structural succession of microbial communities (Di Giacomo et al. 2007; Li et al. 2020; Liu et al. 2022), but little analysis of the air-curing stage has been reported. Air-curing is a key step in differentiating CTLs from roasted tobacco and is a prefermentation treatment for CTLs. To assess the microbial community succession throughout the modulation stage and to predict the function of and explore the microbial interactions, in this study, we harvested CTL samples from four critical periods of air-curing (the yellowing, browning, dry-leaf, and dry-tendon stages) and four fermentation steps and analyzed the fungal and bacterial communities of the tobacco leaves using high-throughput sequencing technology. Further redundancy analysis (RDA) was performed to reveal correlations between microbial composition and changes in enzyme activity and the modulation environment. The pathways of bacterial and fungal microbial communities influencing CTLs were predicted using the SILVA ribosomal RNA database by Tax4Fun and FunGuild in the R package (Liang et al. 2019; Nguyen et al. 2016). Finally, associations among core microbial groups were explored using microbial network correlation analysis (Bastian et al. 2009). The aims of this study were to identify the relevant enzymes and microbiota affecting the modulation process of cigar tobacco, reveal the intrinsic link between the unique flavor and microbial communities during the modulation of CTLs, and provide valuable insights for the improvement of cigar tobacco quality.

## Materials and methods

### Experimental materials

In July 2021, freshly harvested CTLs were strung on bamboo poles and dried at an air-curing room in the natural state for 26 days at a tobacco plantation in Chengjiang County, Yunnan Province. During this period, the temperature and humidity of the air-curing room were monitored. Samples were collected from fresh tobacco leaves (WrC0) and leaves in the yellowing stage (6 days, WrC6), browning stage (12 days, WrC12), dry leaf stage (20 days, WrC20), and dry tendon stage (26 days, WrC26) of air-curing. After air-curing, the CTLs were manually fermented in accordance with the “Yunnan Cigar Tobacco 98 Stacking and Fermentation Technical Regulations” (enterprise standard Q/YNYC(KJ)J 02–2022) and were immediately destacked when the temperature reached 40 °C or exhibited cooling. CTLs were collected from the first stamping (4 days, WrF4), second stamping (10 days, WrF10), third stamping (16 days, WrF16), and fourth stamping (36 days, WrF36) during the fermentation period. Then, the leaves collected during the 9 periods were immediately cut with sterilized scissors. Approximately 20 g samples from between the branches of the same site were loaded in centrifuge tubes, placed in liquid nitrogen for snap freezing, and stored at − 80 °C for subsequent DNA extraction and enzyme activity assays. Three biological replicates were set up for the samples from each time point.

### Measurement of enzyme activity, temperature and humidity

The activities of phenylalanine deaminase (PAL), polyphenol oxidase (PPO), peroxidase (POD), alkaline protease (AKP), amylase (AL), and neutral transforming enzyme (NI) were measured and analyzed at nine time points during the drying and fermentation process. Approximately 0.1 g of each sample was weighed, examined using SolarBio’s micromethod enzyme-linked immunosorbent assay (ELISA) kit, and assayed using an enzyme marker according to the manufacturer’s instructions (Lee et al. 2021), with three biological replicates set up for each sample. The temperature and humidity in the air-curing and fermentation huts during the modulation process were measured and recorded using an electronic probe thermometer and hygrometer.

### DNA extraction and sequencing

The total genomic DNA of the samples was extracted by the hexadecyl trimethyl ammonium bromide (CTAB) method (Su et al. 2011; Zhao et al. 2007), and the purity and concentration of the DNA were determined by 1% agarose gel electrophoresis. Appropriate amounts of sample DNA were placed in a centrifuge tube and diluted to 1 ng/µl with sterile water. Bacterial V4 and fungal ITS1 rRNA regions were amplified using the forward primers 515F (5′-GTGCCAGCMGCCGCGGTAA-3′) and ITS5-1737F (5′-GGAAGTAAAAGTCGTAACAAGG-3′) and reverse primers 806R (5′-GGACTACHVGGGTWTCTAAT-3′) and ITS2-2043R (5′-GCTGCGTTCTTCATCGATGGC-3′), respectively (Yang et al. 2018; Youssef et al. 2009). Polymerase chain reaction (PCR) amplification was performed using New England Biolabs Phusion® High-Fidelity PCR Master Mix with GC Buffer and high-efficiency high-fidelity enzymes. Library construction was performed according to the instructions for the TruSeq® DNA PCR-Free Sample Preparation Kit, the library was sequenced on the Illumina NovaSeq platform, and 250 bp paired-end reads were generated.

### Statistical analyses

PCR amplified sequence data were truncated from barcode and primer sequences and spliced using FLASH (V1.2.7) (Magoč and Salzberg 2011) for each sample read, and the resulting spliced sequences were filtered using fastp software (Bokulich et al. 2013). The chimeric sequences were identified and removed by performing a search (https://github.com/torognes/vsearch/) (Rognes et al. 2016) against the species annotation database (Haas et al. 2011) to obtain the final valid data. Effective tags of the microbiota were clustered using the Uparse algorithm (Uparse v7.0.1001) (Edgar 2013), and sequences were clustered into operational taxonomic units (OTUs) based on 97% identity. Species annotation of the OTU sequences was performed using the Mothur method with the SSUrRNA database (Quast et al. 2013) from SILVA138 (http://www.arb-silva.de/) (Wang et al. 2007), set at a threshold of 0.8 to 1. Rapid multiple sequence alignment was performed using MUSCLE (Edgar 2004) (version 3.8.31) to obtain phylogenetic relationships for all representative OTU sequences. The sample with the lowest amount of data was used as the criterion for homogenization, and subsequent analyses were performed based on the homogenized data. The raw sequencing data have been uploaded to the NCBI Sequence Read Archive (SRA) database under BioProject number PRJNA913282.

### Data analysis

Enzyme activity parameters were analyzed by one-way analysis of variance (ANOVA) using SPSS software (version 25.0.0.2) and visualized by GraphPad Prism9 (version 9.3.0). Alpha diversity and beta diversity were calculated using QIIME software (version 1.9.1) and analyzed using the Wilcox text in the agricolae package in R. Dilution curves were plotted using R software (version 2.15.3) to assess sequencing depth. Dynamic changes and succession of the core microbial community composition were visualized using the ggalluvial package in R (Brunson 2020). Differences between microbial groups were analyzed by PCoA based on Bray‒Curtis distances using the GUniFranc package. Significant taxonomic differences between fungi and bacteria were tested using linear discriminant analysis (LDA) and effect size (LEfSe) analysis. A factorial Kruskal‒Wallis rank sum test (*α* = 0.05) was used to identify groups with significantly different abundances between categories (all-pairs-all comparisons), and then the log LDA score (threshold = 4.0) was used to estimate the effect of each discriminant feature. To analyze the community drivers, redundancy analysis was performed with Canoco 5 (Version 5.02), and significance tests were performed using ANOVA. Functional prediction of bacterial communities was performed using Tax4Fun’s nearest neighbor method based on minimum 16S rRNA sequence similarity (Liang et al. 2019). Functional prediction of fungal communities was performed using the FunGuild database (Nguyen et al. 2016). Correlation networks between microorganisms were constructed using the igraph package in R, and the networks were visualized using Gephi software (Version 0.9.7) based on a correlation matrix constructed by Spearman (Bastian et al. 2009), with each node and edge representing a genus and a strong and significant correlation, respectively.

## Results

### Dynamic changes in CTLs appearance during air-curing and fermentation

**Fig. 1:**
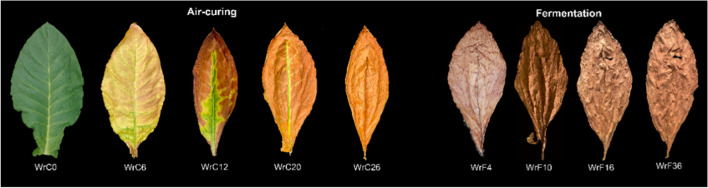
Appearance of CTLs at different periods of the air-curing and fermentation process

As shown in Fig. 1, the change in color of CTLs mainly occurred in the air-curing stage, and the change in toughness and integrity mainly occurred in the fermentation stage. Freshly harvested CTLs showed a dark green color, which faded to yellow during the WrC6 period of the air-curing process. At WrC12, they gradually turned from green to reddish brown from the leaf edge inward, the leaf area shrank, and the flatness of the leaf surface decreased. At WrC20, the green color completely faded except for the main leaf veins. At WrC26, the entire tobacco leaf was consistently uniformly yellowish-brown in color. During the fermentation process, the flatness of the leaf surface gradually decreased, the tobacco leaf gradually changed from grayish brown to brownish brown, and from WrF10 onward, the tobacco leaf showed slight breakage.

### Dynamic changes in enzyme activity during air-curing and fermentation of CTLs

**Fig. 2:**
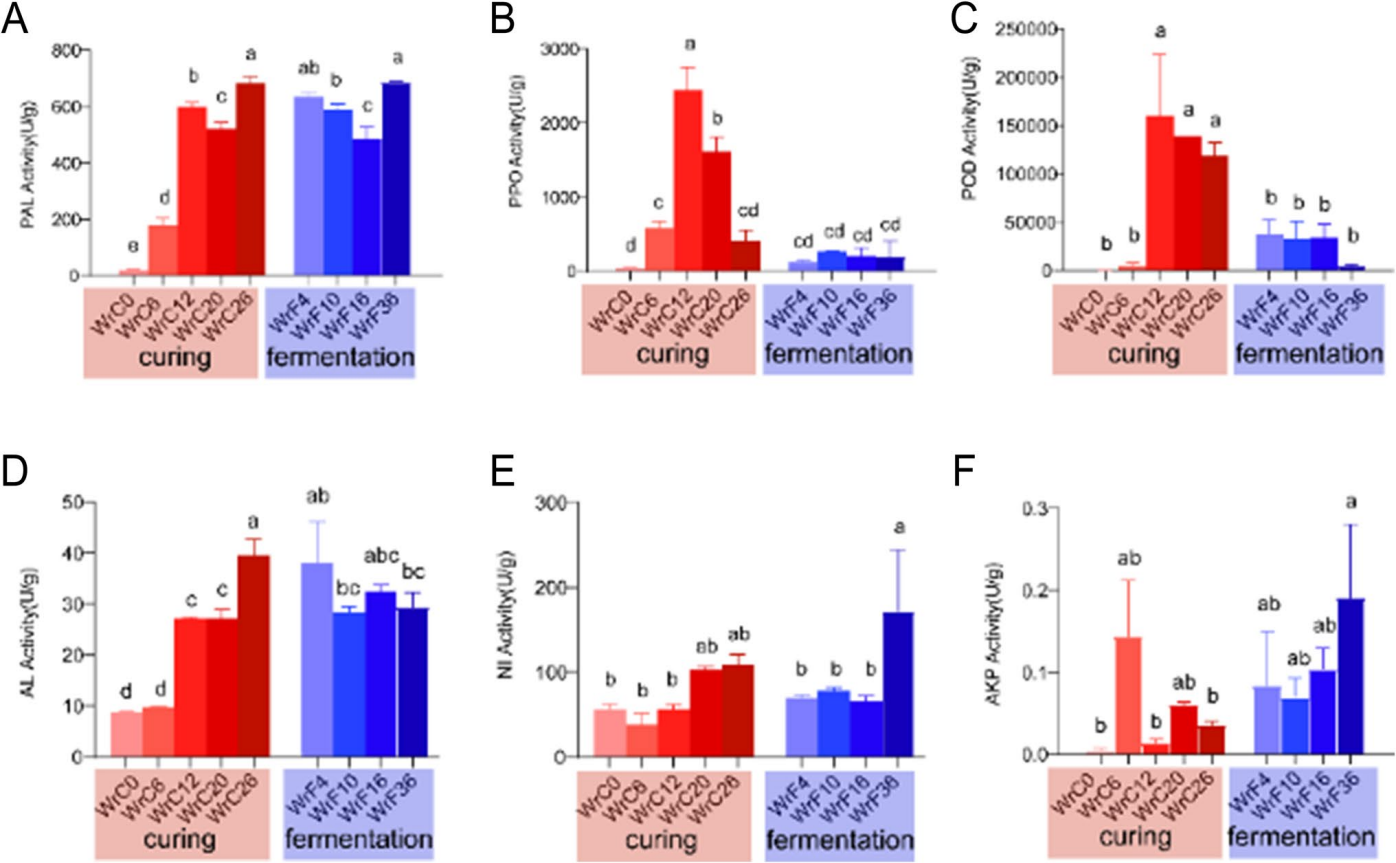
Changes in the activities of phenylalanine deaminase (PAL, **A**), polyphenol oxidase (PPO, **B**), peroxidase (POD, **C**), amylase (AL, **D**), neutral transforming enzyme (NI, **E**), and alkaline protease (AKP, **F**) during air-curing and fermentation of CTLs. The data are expressed as the mean ± SEM with the ANOVA significance test, and changes with different lowercase letters were significant at the 0.05 level (*p* < 0.05). Curing represents the air-curing stage, and fermentation represents the fermentation stage

To find the intrinsic driving factors of quality formation during the air-curing and fermentation processes of CTLs, we measured the specific activity of carbon and nitrogen metabolizing enzymes (AL, AKP, and NI), enzymatic browning-related enzymes (POD and PPO), and polyphenolics synthetase (PAL) during the modulation process. We found that the enzyme activities fluctuated during the air-curing stage and were relatively stable during the fermentation stage (Fig. 2).

Compared to those in fresh tobacco, the activities of PAL, PPO, and POD increased significantly during the air-curing stage, with PAL activity increasing from 19.17 ± 3.27 U/g in fresh tobacco to 178.65 ± 24.42 U/g at the yellowing stage and peaking at 683.86 ± 22.58 U/g in the dry-tendon stage; the PPO activity peaked in the browning stage at 1612.51 ± 195.34 U/g and then gradually decreased to 415.29 ± 142.72 U/g in the dry-tendon stage (Tab. S1). The PAL activity remained at a high level during the fermentation stage, while the activities of PPO and POD decreased to the level in fresh tobacco (Fig. 2A–C). The activity of AL gradually increased during the air-curing stage, reaching a maximum value of 39.54 ± 3.16 U/g during the dry-tendon stage (Fig. 2D). The activities of NI and AKP changed only slightly throughout the modulation process, and the activity of AKP remained low throughout (Fig. 2E, F).

### Dynamic changes in microbial communities during air-curing and fermentation of CTLs

To assess the dynamic patterns of microbial communities during the modulation of CTLs, high-throughput sequencing technology was applied to sequence 27 samples (16S and ITS rRNA). After quality control, the sequencing data were clustered at 97% sequence similarity for OTUs, yielding a total of 11,814 bacterial OTUs and 922 fungal OTUs. The dilution curves for bacteria and fungi tended to be flat (Fig. S1, A, B), with both achieving over 99% coverage (Tab. S2), indicating that deep sequencing provided good overall OTU coverage.

**Fig. 3:**
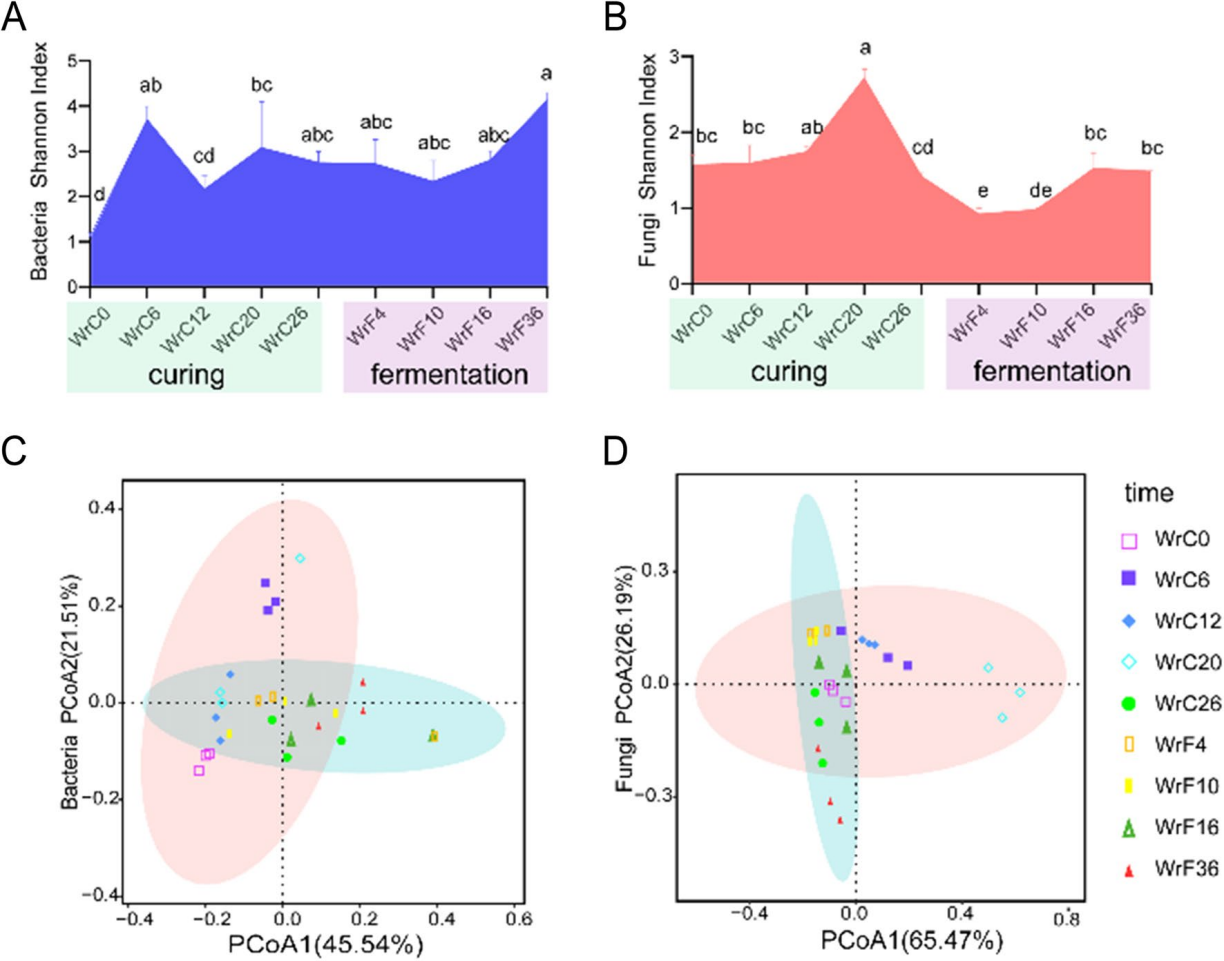
Alpha diversity and beta diversity of microbial communities during modulation of CTLs. **A** Bacterial Shannon index during modulation. **B** Fungal Shannon index during modulation. Changes with different lowercase letters are significant at the 0.05 level (*p* < 0.05). **C** Principal coordinate analysis (PCoA) of bacteria during modulation. **D** PCoA of fungi during modulation

The alpha diversity of the microbial community composition was assessed by the Shannon index, Chao1 index, and Simpson index, and the diversity and richness of the microbial community composition varied with the stage of modulation (Tab. S2). The microbial alpha diversity was significantly higher during the drying phase than during the fermentation phase, but the bacterial alpha diversity peaked at the end of fermentation (Tab. S2). The bacterial community was more diverse than the fungal community, and the dominant bacterial species were more prominent in the community. During air-curing, the bacterial community was more abundant in the yellowing stage, whereas the fungal community was more abundant in the dry-leaf stage; during fermentation, both bacterial and fungal community abundances tended to increase (Fig. 3A, B).

The Bray‒Curtis distance was used to assess the beta diversity of bacteria and fungi. We found that the beta diversity index of bacterial communities was generally higher than that of fungal communities. The bacterial community had the highest beta diversity index during the first stomping period of the fermentation stage, followed by the dry-leaf stage, while the fungal community had the highest beta diversity index during the dry-leaf stage (Fig. S2). PCoA showed significant differences in microbial communities between the air-curing and fermentation stages, but the structure of the WrC26 microbial community composition was more similar to that observed in the fermentation stage (Fig. 3C, D). LEfSe analysis further confirmed that these differences were associated with significant associations between taxa and modulation period (Fig. S3), revealing significant differences in the abundances of 13 bacterial taxa, with five bacterial taxa significantly enriched during the air-curing phase and eight bacterial taxa significantly enriched during the fermentation phase. *Firmicutes*, *Proteobacteria*, and *Bacteroidota* were significantly different between groups and were significantly enriched in WrF36, WrF16, and WrC20, respectively, with *Firmicutes* having the highest abundance (Fig. S3B). LEfSe showed significant differences among the nine fungal taxa, three of which were significantly enriched during the air-curing period and six of which were significantly enriched during the fermentation period, while the LDA scores of *Sordariomycetes* were greater than those of other taxonomic units, indicating that it had a strong influence on the differences between groups (Fig. S3B).

**Fig. 4:**
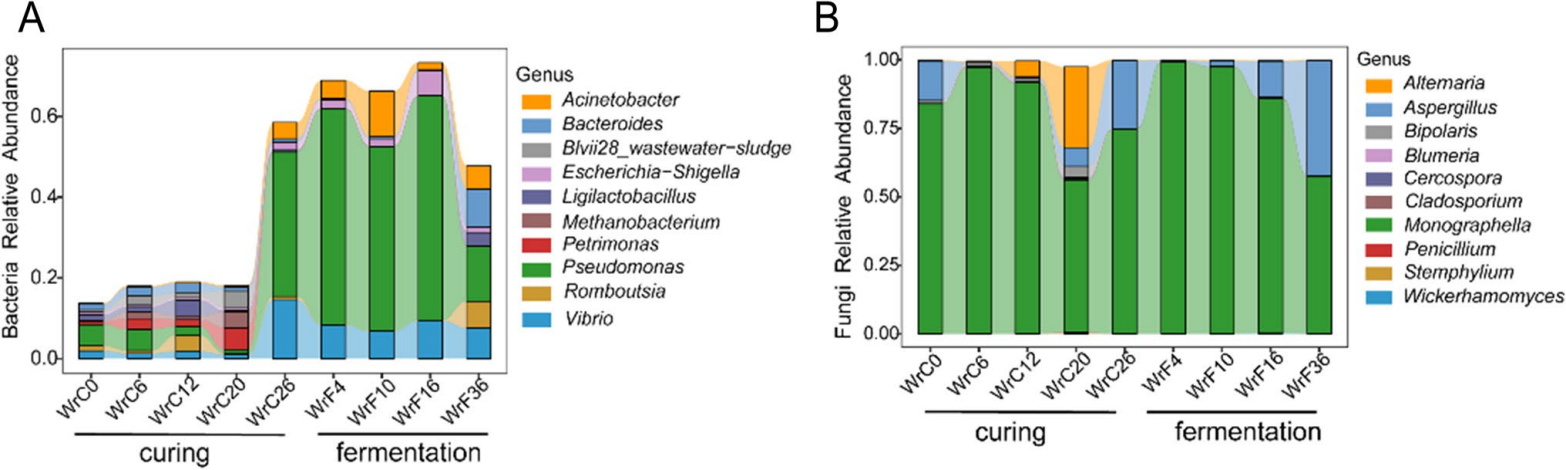
Community dynamics of bacteria (**A**) and fungi (**B**) during modulation of CTLs. The graph represents the top 10 dominant microorganisms in terms of abundance at the genus level

During modulation, the main bacteria observed at the phylum level were *Proteobacteria*, *Firmicutes*, and *Abditibacteriota*; the main fungi observed were *Ascomycota* and *Basidiomycota*. A total of 1086 bacterial genera and 221 fungal genera were detected, with *Pseudomonas*, *Bacteroides*, *Romboutsia*, *Escherichia-Shigella*, *Vibrio*, *Acinetobacter*, *Petrimonas*, *Ligilactobacillus*, *Blvii28 wastewater-sludge group*, and *Methanobacterium* being the main dominant bacterial genera (Fig. S1, C); *Monographella*, *Aspergillus*, *Alternaria*, *Bipolaris*, *Cercospora*, *Wickerhamomyces*, *Cladosporium*, *Stemphylium*, *Penicillium*, and *Blumeria* were the predominant fungal genera (Fig. S1, D). The abundances of *Pseudomonas* and *Vibrio* increased rapidly during WrC26, and these genera played an important role mainly in the air-curing phase and fermentation phase. *Pseudomonas* became the absolute dominant bacterial genus (35.82–55.47%) in the middle of the fermentation period (Fig. 4A). Among the fungal communities, *Monographella* and *Aspergillus* accounted for the vast majority of the modulation throughout, with *Alternaria* abundance increasing from 0.41% at WrC6 to 29.57% at WrC20 before decreasing to 0.22% at WrC26 (Fig. 4B).

### Functional prediction of bacterial and fungal communities during air-curing and fermentation of CTLs

**Fig. 5:**
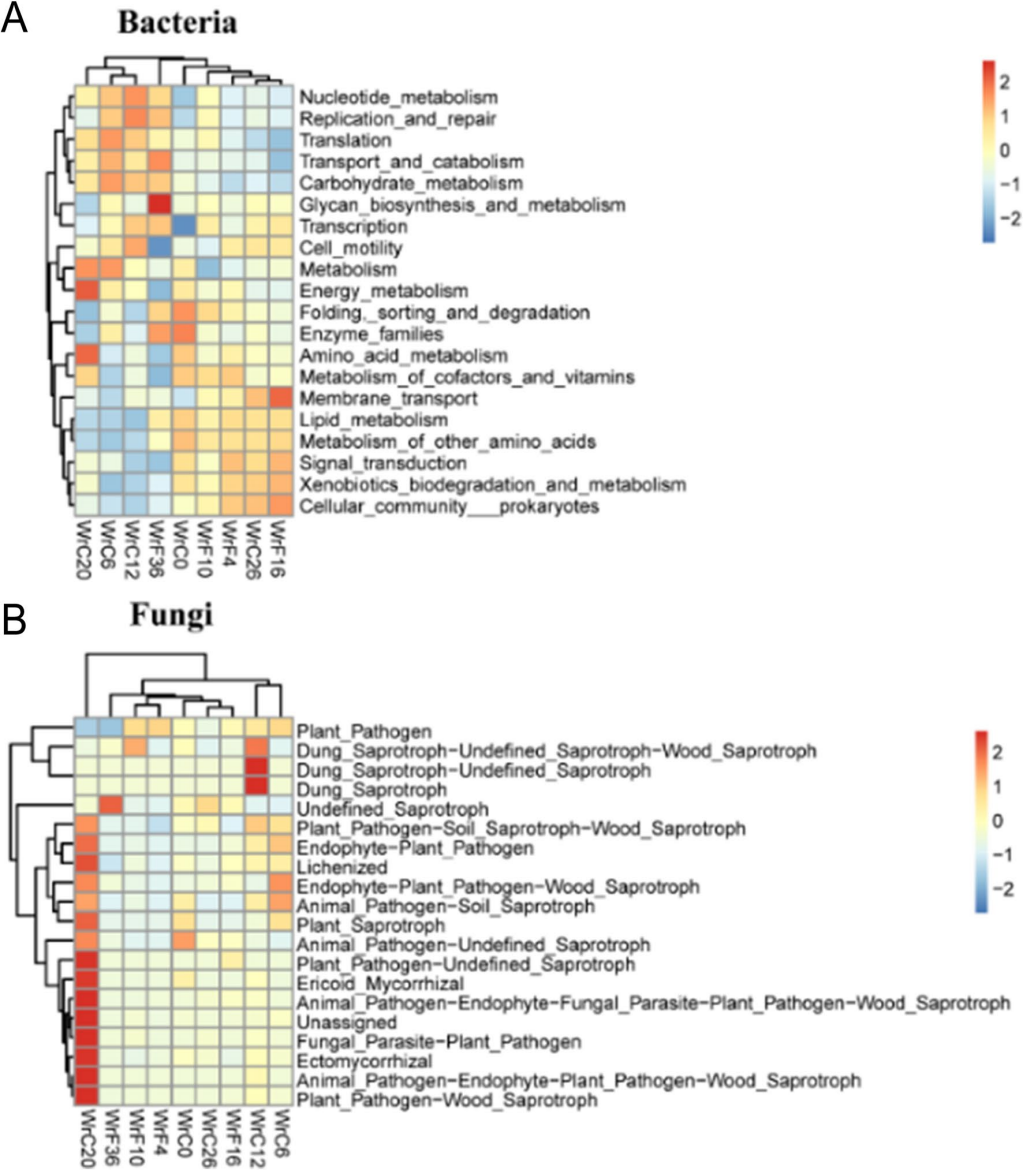
Heatmap of the functional relevance of microorganisms during modulation of CTLs. **A** Functional relevance of dominant bacteria at different modulation stages. **B** Functional correlation of dominant fungi at different stages of modulation

Kyoto Encyclopedia of Genes and Genomes (KEGG) functional enrichment analysis of the bacterial and fungal communities of CTLs during modulation was performed using the Tax4Fun database and FunGuild database, respectively (Liang et al. 2019; Nguyen et al. 2016). We found that the functions of the dominant bacterial community were mainly nucleotide metabolism, carbohydrate metabolism, replication and repair, glycan biosynthesis and metabolism, energy metabolism, amino acid metabolism, etc. The main functions of fungi were dung saprotrophs, plant pathogens, soil saprotrophs, wood saprotrophs, etc. (Fig. 5).

The clustering heatmap showed that the bacterial community was heavily enriched in glycan biosynthesis and metabolic pathways during the WrF36 period. The energy metabolism and amino acid metabolism of bacteria were active during the WrC20 period. The fungal community had soil-rotting and wood-rotting functions mainly during WrC12 and WrC20, and these functions were particularly active during WrC20.

### Interrelationships among enzyme activity, microorganisms, temperature and humidity during air-curing and fermentation of CTLs

**Fig. 6:**
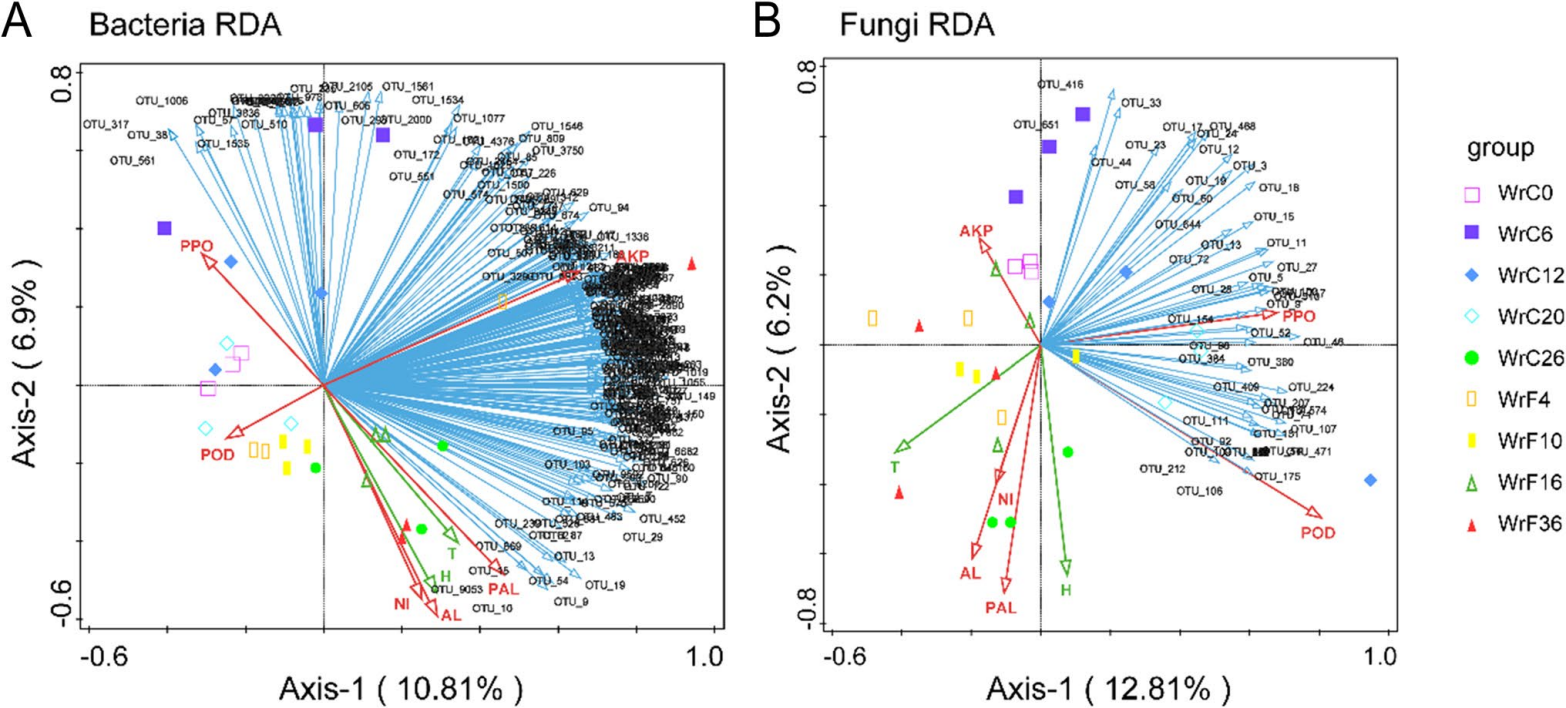
Redundancy analysis (RDA) between enzyme activity and temperature and humidity during air-curing and fermentation of CTLs. The red arrows in the graph represent enzyme activity. The green arrows represent temperature (T) and humidity (H), and the blue arrows represent OTUs that have a contribution

To identify the interactions between enzyme activity, temperature and humidity, and microbial communities, network correlations among enzyme activity, environmental temperature and humidity, and microbial communities were determined by redundancy analysis (RDA) (Fig. 6).

RDA showed that 278 bacterial OTUs and 91 fungal OTUs were correlated with enzyme activity, temperature and humidity. These bacterial OTUs were classified at the phylum level mainly as *Proteobacteria*, *Firmicutes*, *Bacteroidota*, and *Acidobacteriota* and at the family level mainly as *Lachnospiraceae*, *Rhodobacteraceae*, *Acetobacteraceae*, *Comamonadaceae*, *Muribaculaceae*, and *Bacteroidaceae*. These fungal OTUs were classified at the phylum level as *Ascomycota* and *Basidiomycota* and at the family level mainly as *Hyponectriaceae*, *Pleosporaceae*, *Trichocomaceae*, and *Nectriaceae*.

PPO mainly influenced the yellowing and browning periods in the air-curing stage and was negatively correlated with NI, AL, and PAL. There was a positive correlation among NI, AL, and PAL; they played an important role mainly at the end of fermentation. AKP was positively correlated with the bacterial community and negatively correlated with POD, and PPO was positively correlated with the fungal community. Ambient temperature and humidity mainly affected the fermentation process, especially at the end of fermentation. The ambient temperature was negatively correlated with the fungal community during the fermentation process, while the relationship with the bacterial community was more complex.

### Overview of the correlation network among microorganisms during air-curing and fermentation of CTLs

**Fig. 7:**
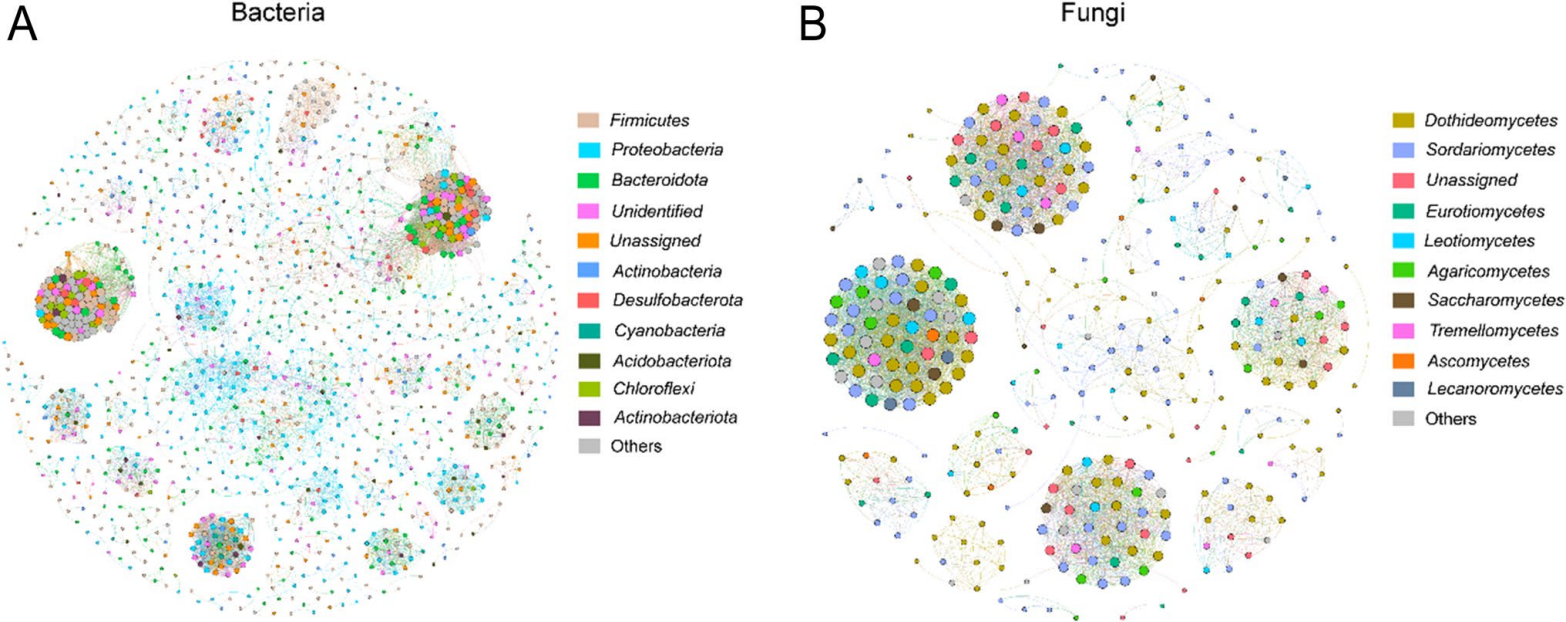
Spearman’s correlation analysis of bacteria (**A**) and fungi (**B**) during air-curing and fermentation of CTLs. The circles in the graph represent OTUs, with larger circles representing stronger correlations and different colors representing OTUs classified in different orders. The correlation *R* > 0.8, and the graph indicates a significant change at the 0.05 level (*P* < 0.05)

**Fig. 8:**
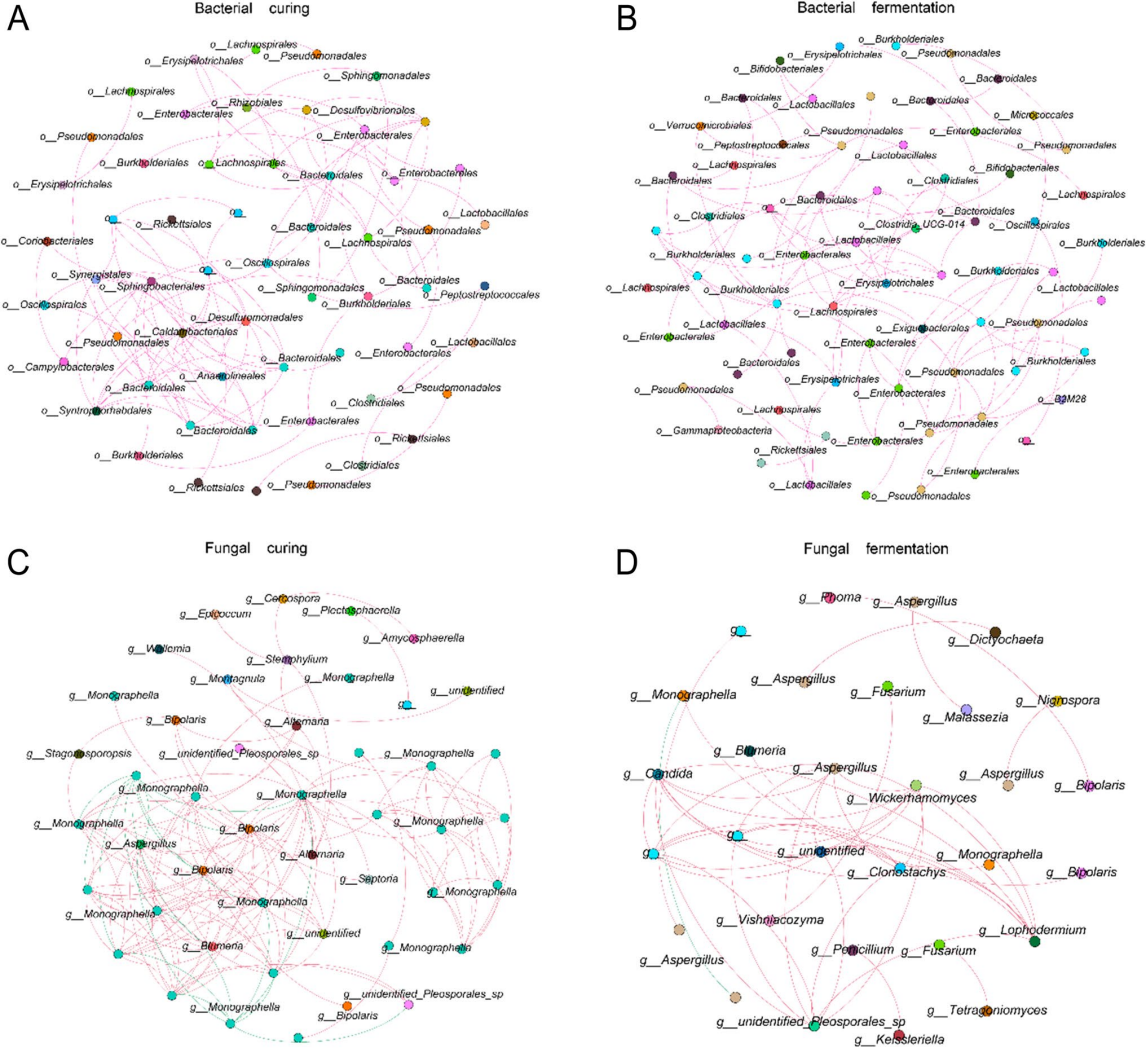
Spearman’s correlation analysis plot between CTL microorganisms during air-curing and fermentation. The graphs indicate the correlation networks between the bacterial air-curing (**A**) and fermentation (**B**) stages and fungal air-curing (**C**) and fermentation (**D**) stages. The red line indicates a positive correlation, and the green line indicates a negative correlation. Circles indicate OTUs, text indicates OTU classification at the genus level, and circles of the same color indicate OTU annotations in the same genus. The correlation *R* > 0.9, and the graphs indicate a significant change at the 0.01 level (*P* < 0.01)

To explore microbial interactions, we mined 16S vs. ITS sequencing data using Spearman correlation network analysis, which revealed that the network correlations among microbes were all predominantly positive, with the complexity of interactions between bacteria being significantly greater than those between fungi (Figs. 7 and 8). At the bacterial phylum level, there were significant positive correlations among *Firmicutes*, *Proteobacteria*, *Actinobacteria*, *Desulfobacterota*, and *Bacteroidetes* (Fig. 7A); at the fungal class level, there were significant positive correlations among *Dothideomycetes*, *Sordariomycetes*, *Eurotiomycetes*, *Leotiomycetes*, and *Agaricomycetes* (Fig. 7B). The interactions among the dominant microorganisms in the air-curing and fermentation stages of the modulation process were significantly different (Fig. 8).

## Discussion

Enzyme activity and the microbial community control the modulation process of CTLs, and microbial regulation of CTLs is achieved mainly through the biosynthesis and catabolism of metabolic enzymes (Zhang et al. 2021). Proper modulation is the main reason for the unique taste and aroma of cigars (Zhao et al. 2009). To solve various problems such as high irritation, green taste, and lack of aftertaste in domestic cigar smoking, there is an urgent need to explore the function of the main role of enzyme activity and microbial community in the air-curing and fermentation stages. For this purpose, we analyzed the enzymatic activity and microbial diversity of CTLs at nine time points during the modulation phase.

The trends in the appearance, enzyme activity, and microbial diversity of CTLs indicated that the microbial community succession processes and metabolic functions changed significantly during the air-curing phase, and the effect of the fermentation phase was continuous and moderate. PCoA and microbial Spearman correlation analysis showed that there were significant differences in the dominant microbial community interactions between the air-curing and fermentation phases. The bacterial community was more diverse, complex, and diversified than the fungal community and had a greater influence on the air-curing and fermentation processes of CTLs.

The enzyme activities of CTLs during the conditioning process were generally higher than those of fresh tobacco, indicating that in addition to the enzymes of the tobacco leaves themselves, the enzymes secreted by microorganisms also played an important regulatory role. PPO mainly affected the WrC6 and WrC12 periods during the air-curing stage (Fig. 6), where the enzyme activities also showed an increasing trend and reached a maximum level during the WrC12 period (Fig. 2B). PPO and POD control the breakdown of polyphenols (Chen et al. 2019) and improve the appearance and quality of tobacco by acting on polyphenols to produce browning, but excessive oxidation and enzymatic browning can darken the color of tobacco (MATHEIS and Whitaker 1984; Piyada et al. 2007). Polyphenols have strong aromatic properties (Wang et al. 2018), and PAL catalyzes the conversion of phenylalanine to polyphenols, resulting in the accumulation of aromatic components in CTLs. During the period from WrC6 to WrC12, CTLs gradually turned brown from the leaf margin inward, and then the color stabilized, which was consistent with the change trend of PPO. We found that PPO is the key enzyme for enzymatic browning. Apparently, the brown color production of CTLs during the period from WrC6 to WrC12 was related to the enzymatic browning controlled by PPO. High-grade CTLs, especially those of wrapping tobacco, tend to have a greater demand for color and appearance than for smoking taste. Controlling the dominant microbial community composition to regulate PPO activity within a certain range can improve the appearance of CTLs (Chen et al. 2019; Piyada et al. 2007). The activity of AKP remained low throughout (Fig. 2F), probably due to the low pH during the modulation process (Sharma et al. 2017).

The dominant microorganisms during the fermentation period may increase the accumulation of small-molecule sugars by secreting the corresponding enzymes that breakdown starch and sugars (Ezquer et al. 2010; Wu et al. 2021). NI, AL, and PAL stabilized at high levels of enzyme activity during the fermentation phase (Fig. 2), and Fig. 6 shows that NI, AL, and PAL play an important role mainly at the end of the fermentation process. Similarly, bacterial community glycan biosynthesis and metabolism was actively functioning during the WrF36 period. Excess starch in tobacco leaves can lead to a burnt smell during smoking, and high starch and nicotine levels can lead to coarse smoke and accompanying irritation (Zhao et al. 2012, 2009). Carbon and nitrogen metabolism breaks down nicotine, protein, starch, and sugars to smaller molecules (Banožić et al. 2020), AL can breakdown starch into small molecules of sugars and reduce the burning odor of starch, and proper AL can improve the burnability of cigars and reduce the bitter and harsh taste (Ezquer et al. 2010). Overall, the results indicate that although changes such as microbial community and enzyme activity mainly occur in the air-curing stage, carbon metabolism and the formation of polyphenols play an important role in the fermentation stage.

During the WrC20 period, the energy metabolism and amino acid metabolism of the bacterial community were active, indicating that the dominant bacterial community started a massive growth and reproduction process at this time. At the same time, the highest beta diversity index of bacteria was observed at this stage, indicating the greatest degree of change in species diversity at this time. The role of fungi in the modulation of CTLs may be associated with the degradation of lignin, cellulose, and pectin (Su et al. 2016). The saprophytic effect of the fungal community was observed mainly during the air-curing period, with a strong saprophytic effect detected at WrC12 and WrC20, especially at WrC20 (Fig. 5B), indicating that WrC20 is a critical period for the degradation of lignin, cellulose, pectin, etc. Furthermore, the abundance of *Alternaria* rapidly increased from 0.41% in WrC6 to 29.57% in WrC20, while intense putrefaction occurred, and it is reasonable to suspect that the increase in abundance of *Alternaria* is the possible cause of the intense putrefaction; however, this needs to be further verified (Figs. 4 and 5).

Ambient temperature and humidity during modulation affect the growth and metabolism of microorganisms (Zhao et al. 2022). Ambient temperature and humidity mainly affect the fermentation process, where ambient temperature is negatively correlated with the fungal community of the fermentation process, while the relationship with the bacterial community is more complex (Fig. 6). During the fermentation process, CTLs generate a large amount of heat internally that cannot be released due to the stacking effect. To ensure uniform heating, the stacking process is often carried out at intervals during the fermentation process. The stacking process and the microbial community during the fermentation stage are clearly correlated.

At the phylum level, the dominant bacteria observed were *Proteobacteria*, *Firmicutes*, and *Abditibacteriota*, and the dominant fungi were *Ascomycota* and *Basidiomycota*. These microorganisms were also associated with enzymes and temperature and humidity, suggesting that these dominant microbial communities in the formation of CTLs quality These dominant microbial communities play an important role in the formation of CTLs quality. *Pseudomonadales* have the ability to degrade nicotine, and some *Pseudomonadales* have the ability to breakdown amino acids and lignocellulose (Huang et al. 2020; Zhang et al. 2022). *Enterobacterales* have the ability to breakdown proteins and amino acids. Of course, the role among microorganisms is not singular but is regulated by a complex network of interactions among microbial communities (Fig. 8). Modulation processes improve the intrinsic quality of CTLs through the combination of multiple microbial communities (Abisado et al. 2018; Gralka et al. 2020; Ma et al. 2021; Tashiro et al. 2013).

This study revealed the microbial diversity and composition during the preparation of cigar tobacco by high-throughput sequencing technology. The microbial community composition dynamics during air-curing and fermentation played an important role in improving the quality of cigars. The alpha diversity of microorganisms was significantly higher in the air-curing stage than in the fermentation stage, with *Pseudomonas*, *Bacteroides*, *Vibrio*, *Alternaria*, *Proteobacteria*, *Monographella*, *Bipolaris*, and *Aspergillus* playing an important role in the improvement of cigar quality. PPO and POD play a key role in the color of tobacco in the air-curing stage, while carbon- and nitrogen-related enzymes are more relevant to the microbial community composition in the fermentation stage. Bacteria improved the quality of CTLs mainly through sugar metabolism, amino acid metabolism, and lipid metabolism, while fungi improved the levels of lignin, cellulose, and pectin mainly through saprophytic action. Among bacteria, *Firmicutes*, *Proteobacteria*, *Actinobacteria*, and *Bacteroidota* play important roles in determining cigar quality; fungi are mainly *Dothideomycetes*, *Sordariomycetes*, *Eurotiomycetes*, *Leotiomycetes*, and *Agaricomycetes*. Exploring the relationship between the key microbial community composition and the metabolic enzymes in the air-curing and fermentation processes can provide a reference for improving the drying and fermentation processes of CTLs and provide information for improving cigar quality.

## Supplementary Information

Below is the link to the electronic supplementary material.

Supplementary file1 (PDF 656 KB)

## Data Availability

The datasets generated during and/or analyzed during the current study are available from the corresponding author on reasonable request.
