# Supplementary material for: Microbial and enzymatic changes in cigar tobacco leaves during air-curing and fermentation
Source: Appl Microbiol Biotechnol. 2023 Jul 17;107(18):5789–801. doi: 10.1007/s00253-023-12663-5 (PMC10439857; doi:10.1007/s00253-023-12663-5)
Supplement: Supplementary file 1 — Supplementary file1 (PDF 656 KB) [file 253_2023_12663_MOESM1_ESM.pdf]

Supplementary Material

Journal Name: Applied Microbiology and Biotechnology

Article Title: Microbial and enzymatic changes in cigar tobacco leaves during air-curing and fermentation

Authors: Qing Zhang, Guanghui Kong, Gaokun Zhao, Jun Liu, Honggang Jin, Zhihua Li, Tao Liu*, Guanghai Zhang*

*** Correspondence:**

Guanghai Zhang, zghzxf@126.com, Yunnan Academy of Tobacco Agricultural Sciences, Kunming, Yunnan, 650021, China

Tao Liu, yantao618@126.com, College of Agriculture and Biotechnology, Yunnan Agricultural University, Kunming, Yunnan, 650201, China

Fig. S1 Dilution curves and microbial abundance of fungi and bacteria during air-curing and fermentation. A: bacterial dilution curve; B: fungal dilution curve; C: bacterial abundance; D: fungal abundance

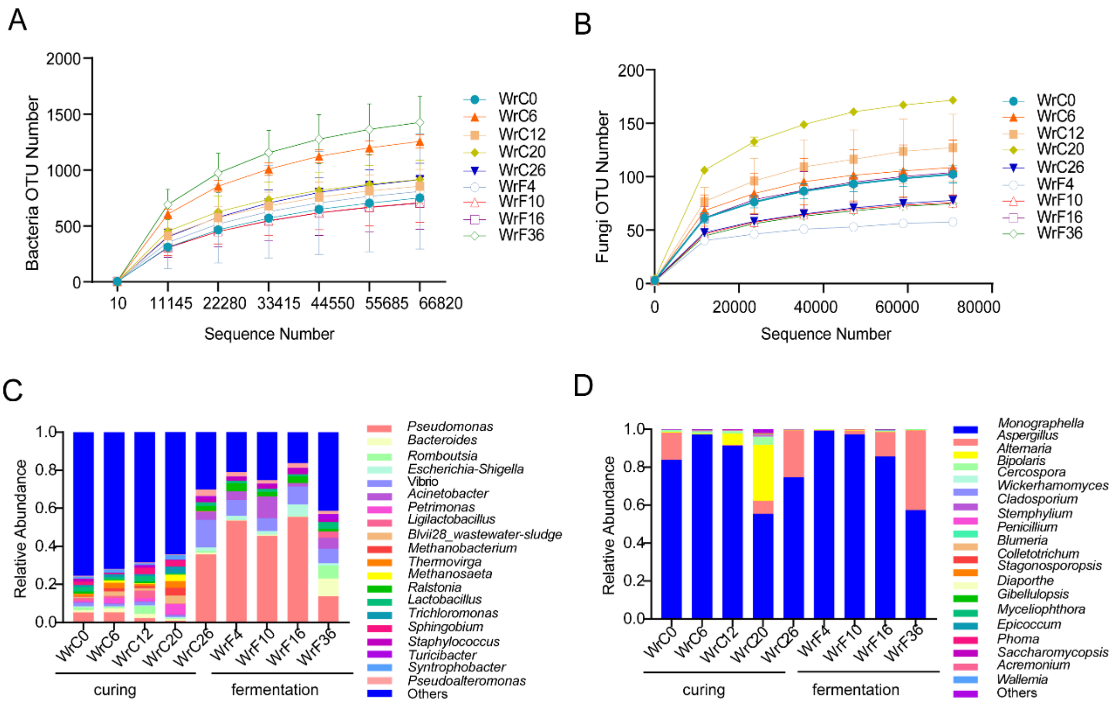

Fig. S2 Box line plot of the beta diversity index.

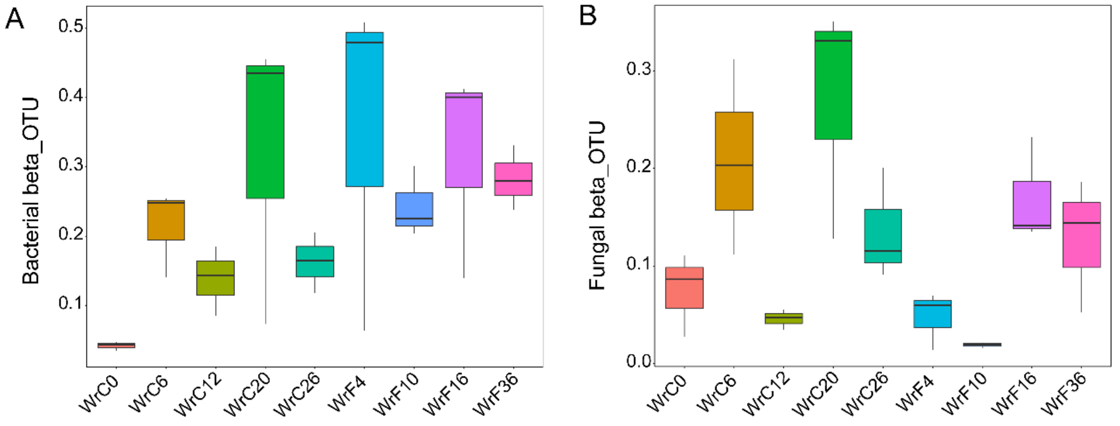

Fig. S3 LEfSe analysis of bacteria (A) and fungi (B) during air-curing and fermentation.

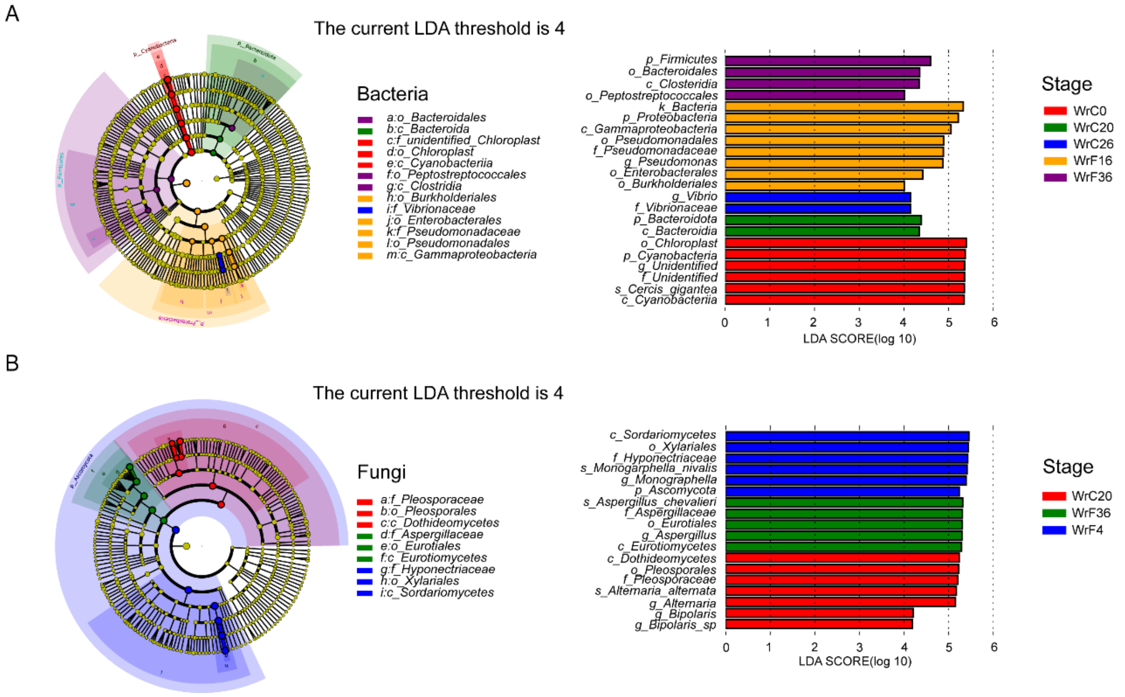

Table S1 Changes in the activities of PAL, PPO, POD, AL, NI and AKP during modulation of CTLs.

Stage	PAL	PPO	POD	AL	NI	AKP
WrC0	19.17±3.27c	41.57±7.95d	489.93±189.81b	8.7±0.29d	56.02±7.07b	0.004±0.005b
WrC6	178.65±24.42d	580.79±96.18c	4489.48±3486.47b	9.79±0.18d	37.87±13.61b	0.143±0.118ab
WrC12	599±14.75b	2443.3±308.44a	160479.43±63209.79a	27.13±0.37c	266.52±214.7b	0.014±0.008b
WrC20	521.69±21.92c	1612.51±195.34b	138765.53±63.96a	27.1±1.84c	102.71±4.27ab	0.060±0.007ab
WrC26	683.86±22.58a	415.29±142.72cd	117914.38±24537.97a	39.54±3.16a	108.87±12.43ab	0.035±0.008b
WrF4	636.26±14.11ab	118.9±40.67cd	36806.77±16202.38b	38.01±8.21ab	69.9±3.48b	0.084±0.116ab
WrF10	590.77±18.08b	270.96±7.11cd	32500.59±18022.69b	28.46±1.02bc	77.7±4b	0.070±0.040ab
WrF16	487.09±41.47c	203.79±106.48cd	33427.1±14580.69b	32.49±1.27abc	65.29±7.09b	0.104±0.044ab
WrF36	687.24±5.52a	191.9±128.65cd	4454.9±1112.05b	29.43±1.68bc	171.69±72.01a	0.191±0.156a

Table S2 Alpha diversity indices of bacteria and fungi during modulation of CTLs.

	Bacterial diversity				Fungal diversity			
Sample	Shannon	Chao1	Simpson	Coverage (%)	Shannon	Chao1	Simpson	Coverage (%)
WrC0	1.12	789.34	0.21	99.8	1.57	96.53	0.49	100
WrC6	3.71	1289.40	0.71	99.7	1.59	104.35	0.51	100
WrC12	2.16	875.55	0.42	99.8	1.74	118.48	0.52	100
WrC20	3.08	921.92	0.58	99.8	2.72	164.58	0.73	100
WrC26	2.75	932.69	0.58	99.7	1.42	72.50	0.50	100
WrF4	2.72	816.12	0.62	99.8	0.92	54.43	0.27	100
WrF10	2.34	713.34	0.53	99.8	0.98	72.79	0.28	100
WrF16	2.80	727.09	0.66	99.8	1.53	98.65	0.48	100
WrF36	4.14	1452.00	0.75	99.7	1.49	67.91	0.56	100
